# Patterns and Determinants of Multimorbidity in Older Adults: Study in Health-Ecological Perspective

**DOI:** 10.3390/ijerph192416756

**Published:** 2022-12-14

**Authors:** Yiming Chen, Lei Shi, Xiao Zheng, Juan Yang, Yaqing Xue, Shujuan Xiao, Benli Xue, Jiachi Zhang, Xinru Li, Huang Lin, Chao Ma, Chichen Zhang

**Affiliations:** 1 School of Health Management, Southern Medical University, Guangzhou 510515, China; 2 Department of Health Management, Shunde Hospital, Southern Medical University, Foshan 528399, China; 3 School of Health Management, Bengbu Medical College, Bengbu 233030, China; 4 Department of Health Management, Nanfang Hospital, Southern Medical University, Guangzhou 510515, China; 5 Institute of Health Management, Southern Medical University, Guangzhou 510515, China

**Keywords:** multimorbidity, older adults, pattern, health-ecological model, health management

## Abstract

(1) Background: Multimorbidity has become one of the key issues in the public health sector. This study aims to explore the patterns and health-ecological factors of multimorbidity in China to propose policy recommendations for the management of chronic diseases in the elderly. (2) Methods: A multi-stage random sampling method was used to conduct a questionnaire survey on 3637 older adults aged 60 and older in Shanxi, China. Association rule mining analysis (ARM) and network analysis were applied to analyze the patterns of multimorbidity. The health-ecological model was adopted to explore the potential associated factors of multimorbidity in a multidimensional perspective. A hierarchical multiple logistic model was employed to investigate the association strengths reflected by adjusted odds ratios and 95% confidence. (3) Results: Multimorbidity occurred in 20.95% of the respondents. The graph of network analysis showed that there were 6 combinations of chronic diseases with strong association strengths and 14 with moderate association strengths. The results of the ARM were similar to the network analysis; six dyadic chronic disease combinations and six triadic ones were obtained. Hierarchical multiple logistic regression indicated that innate personal traits (age, history of genetics, and body mass index), behavioral lifestyle (physical activity levels and medication adherence), interpersonal network (marital status), and socioeconomic status (educational level) were the common predictors of multimorbidity for older adults, among which, having no family history was found to be a relative determinant as a protective factor for multimorbidity after controlling the other covariates. (4) Conclusions: multimorbidity was prevalent in older adults and most disease combinations are associated with hypertension, followed by diabetes. This shows that diabetes and hypertension have a high prevalence among older adults and have a wide range of associations with other chronic diseases. Exploring the patterns and associated factors of multimorbidity will help the country prevent complications and avoid the unnecessary use of the health service, adopting an integrated approach to managing multimorbidity rather than an individual disease-specific approach and implementing different strategies according to the location of residence.

## 1. Introduction

Rapid aging and greater longevity have become a global issue, and the elderly population is increasing globally and speedily [1,2]. According to the data reported by the National Bureau of Statistics of China, 18.7% of the total population were aged 60 years and above in 2020, and this number is expected to rise to over 4.3 billion in 2050 [3,4]. An increase in chronic diseases usually accompanies rapid aging; the number of people with multimorbidity is expected to rise at a rate of more than 1% per year by 2030 as the population ages [5], escalating the burden of multimorbidity on individuals and health care systems because people with chronic diseases are more likely to have adverse health outcomes and greater healthcare needs [1,6,7]. The challenges require a radical shift from disease-specific research to a more holistic view of our health. Therefore, studies focused on multimorbidity are being increasingly emphasized [8].

Multimorbidity, defined as the co-occurrence of at least two chronic diseases in the same person [9], is increasingly recognized as a critical public issue. A systematic review stretching across low-, middle-, and high-income countries found that the prevalence of multimorbidity increases with age [10]. The prevalence of multimorbidity among Chinese people aged 50–59 years is 33.8%, while 56.5% for those aged 80 years and above [11,12]. Moreover, multimorbidity patterns have gradually developed into being a trendy topic of current studies. Previous research on the prevalence of multimorbidity and its patterns may not be comparable due to variances in the study population, coding systems, eligible diseases, and analytical methods [13,14,15]. Garin et al. classified the typical patterns of multimorbidity into three categories: the “cardiopulmonary-respiratory” pattern (angina, asthma, and COPD), “metabolic” pattern (diabetes, obesity, and hypertension), and “mental-articular” (arthritis and depression) patterns [10]. Different patterns of multimorbidity can recognize the common risk factors, pathogenesis, and drug interactions among chronic diseases [16,17]. Zhang et al. identified 24 chronic disease association rules for the rural and urban elderly, which proved that hypertension almost existed in each chronic disease combination [7].

The pathogenesis of multimorbidity is complicated, so it is necessary to analyze the prevalence, patterns, and potential associated factors of multimorbidity in older adults. The well-established associated factors are biological and behavioral risk factors (e.g., elderly, female, overweight, smoking, drinking, and living alone) [9,18,19,20]. Likewise, various societal factors (e.g., household income, occupation, education, and living in a deprived area) are associated with multimorbidity [21,22,23]. However, previous studies on the factors underlying multimorbidity in older adults mainly focused on individual-level factors, and the multilevel complexity of multimorbidity was ignored [24,25,26]. To reflect this complexity, some studies have introduced cognitive psychological theories as a basis for factor screening, but few have involved comprehensive contextual characteristics [27]. For example, Ingram et al. assessed the association between household and area-level factors and multimorbidity based on the social determinant of a health model. However, they ignored the role of social and cultural contexts [21]. This leads to the risk of missing correlated factors that may account for more variation in the results and exaggerate the effect of the observed characteristics [28].

To address the current gaps in the literature, this study introduces the health-ecological model, which was developed from the socio-ecological model, to explore the impact of varying dimensions of factors on health outcomes. The socio-ecological model, constructed by Bronfenbrenner in 1977 [29], proposed that the health outcome is affected by a range of variables at different levels: the individual, behavioral, interpersonal, community, and policy environment [30]. Framing associated factors within a health-ecological framework provide a precise understanding of the complexity of determining mechanisms and a micro and macro perspective on health management [27]. Given the complexity of most public health challenges, the health-ecological model has been increasingly embraced in the field of public health [31]. Numerous studies have applied the health-ecological model to understand complex public problems, such as mental health in the elderly [32], cancer screening adherence [33], and health disparities [34]. However, fewer literatures have used this model to study the underlying factors of multimorbidity. Therefore, this study sought to quantify the prevalence and patterns of multimorbidity among older adults in Taiyuan, a northern city in China, and then analyze the potential factors associated with multimorbidity based on the health-ecological model, offering practical implications for comprehensive health management measures. We hypothesized that the prevalence of multimorbidity is higher in older adults. Multimorbidity is influenced by individual congenital, behavioral, family and social networks, and socio-economic and macro-environmental characteristics.

## 2. Materials and Methods

### 2.1. Study Design and Participants

A questionnaire-based, cross-sectional health survey of older adults residing within Taiyuan, the capital city of Shanxi province, was conducted, concluding six districts. In order to get a representative sample of the older adults, a multi-stage stratified cluster sampling method was used in this study to increase the precision, reduce the costs, and reduce the occurrence of a non-response. The sampling method was as follows: first, each street/town was numbered according to the administrative order of Taiyuan city. Next, two community/administrative villages were randomly selected using the random number table from each selected street/town. Finally, two residential districts/natural villages were drawn from each district in the same way. Older adults in residential communities/natural villages who met the criteria were selected as the study population.

The inclusion criteria for this survey were: (1) participants aged 60 years and above; (2) being conscious and able to communicate in Chinese; and (3) voluntarily participating in this study. Older adults were excluded from the study if they had severe cognitive impairment or severe illness and a poor compliance.

All the respondents were interviewed face-to-face using a structured questionnaire by trained interviewers with medical knowledge. We used face-to-face interviews rather than self-filling questionnaires since some older people are illiterate and some are unable to read or write due to poor vision, hand tremors, or other reasons. All the participants were informed of the purpose and procedures of the study upon their recruitment. A total of 3800 participants were invited to answer a standardized questionnaire in this sample, and 3637 older adults completed this survey. The effective questionnaire response was 95.71% (3637/3800). All the study procedures were approved by the Shanxi University Ethics Committee.

### 2.2. Variables

#### 2.2.1. Outcome Variable

To provide an effective analysis of the prevalence, patterns, and determinants of multimorbidity, we principally assessed the number of chronic diseases used in defining multimorbidity in the questionnaire. By default, at least 12 kinds of chronic conditions and six chronic condition categories are required to ensure the accuracy of the results [35,36]. Therefore, according to the previous literature [36,37], the prevalence of chronic diseases in Shanxi province [38], and the suggestions of clinicians, 24 chronic diseases were selected, which can be seen in Supplementary materials.

#### 2.2.2. Independent Variable

**Figure 1:**
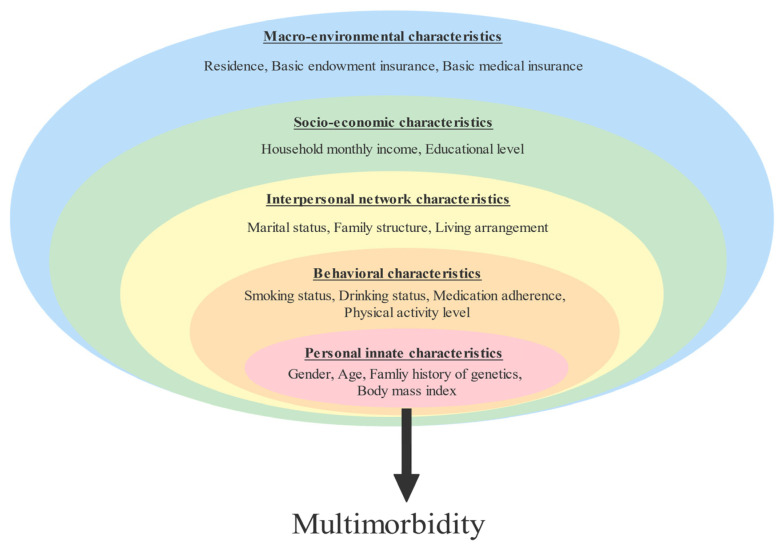
Health ecological model of multimorbidity.

The questionnaire consisted of three sections: general information, lifestyle behavior, and chronic disease status. As shown in Figure 1, the factors affecting older adults with multimorbidity were integrated into a multi-level health-ecological model (e.g., personal innate-, behavioral-, family and social network-, socioeconomic and macro-environmental characteristics) in this study. The prevalence of a chronic disease was determined by whether the respondents had chronic diseases and what kind of chronic diseases they had within six months before the survey. The presence or absence of chronic diseases was collected by the self-report of the respondents, and a precise case diagnosis from a hospital at the county level or above was shown.

### 2.3. Statistical Analysis

All the data were statistically analyzed using an IBM SPSS 22.0 and SPSS Modeler. According to the outcome variable, the categorical variables were presented as numbers and percentages, using a Pearson χ^2^ trend test or Fisher exact test to compare the prevalence of multimorbidity among the elderly with diverse chronic disease types and demographic characteristics.

In the network analysis, each chronic disease is shown as a dot. If a person has two chronic diseases at the same time, the points of the two chronic diseases are connected by a segment of a line. The thickness of the line segments demonstrates the strength between two linked chronic diseases. A higher strength indicates a higher risk of being diagnosed with the associated disease. Therefore, different types of lines can roughly reflect the strength of the correlation. Dashed lines indicate weak correlations; regular thin and thick lines represent moderate and robust correlations, respectively.

Association rule analysis was applied to analyze the pattern of multimorbidity in the elderly by extracting valuable disease pairs as association rules in extensive, disordered data. It is indispensable to discover close correlations between items based on a large amount of data. As one of the most classical algorithms of association rules [39], the ARM can help researchers extract valuable knowledge from massive data sets as a data mining technique. Measurement ratios include Support, Confidence, and Lift. Support (S) A→B denotes the probability that A and B occur together. Confidence (C) is the conditional probability of the occurrence of the consequent, given the antecedent. Lift (A) is the ratio of the observed support to that expected if A and B were independent. Based on this study, the support of A→B was the probability of the simultaneous occurrence of chronic diseases A and B. The confidence was the conditional probability of suffering from chronic disease B under the premise of suffering from chronic disease A. The degree of lift reflects the influence of the consequent B on the antecedent A compared to the overall. Hence, the degree of Lift A→B > 1 indicates that chronic disease A and B have a directional association. In the study, we set the minimum conditional support to 1.5%, the minimum rule confidence to 30%, and the maximum number of preceding items to five.

A hierarchical multiple logistic regression model was employed to examine the relationships between the potential associated factors and multimorbidity. Five hierarchical levels were used in this study. The independent variables were grouped into five hierarchical blocks according to the health-ecological model and were entered with the simultaneous forced entry method in the regression model by Block 1 (personal innate characteristics), Block 2 (behavioral characteristics), Block 3 (family and social network), Block 4 (socioeconomic characteristics), and Block 5 (macro-environmental characteristics). Odds ratios (ORs) and 95% confidence intervals (CIs) were calculated. All the tests were two-tailed, and the significance level was set at 0.05.

## 3. Results

### 3.1. The Prevalence of Chronic Disease and Multimorbidity in the Elderly

A total of 3637 participants were included in this study, in which nearly 51.3% were male and 48.7% were female, with the average age being 70.49 ± 11.35 years. Among the 3637 participants recruited for the study, 2504 (68.85%) reported suffering from chronic disease. Of these, 1742 (47.90%) had simple chronic disease and 764 (20.95%) had multimorbidity. The prevalence of chronic disease and multimorbidity are shown in Table 1. Based on the prevalence of chronic disease, 38.7%, 13.2%, and 6.2% of older adults had probable hypertension (HTN), diabetes mellitus (DM), and rheumatoid arthritis, respectively. Of the 764 patients with multimorbidity, 522 (14.4%) had two chronic diseases, 167 (4.6%) had three chronic diseases, and 73 (2.0%) had four or more chronic diseases at the same time. Notably, the disease with the highest proportion of multimorbidity was osteoporosis (65.0%), followed by eye disease (65.0%) and coronary artery disease (CAD) (61.0%).

### 3.2. Patterns of Multimorbidity

#### 3.2.1. Network Analysis

**Figure 2:**
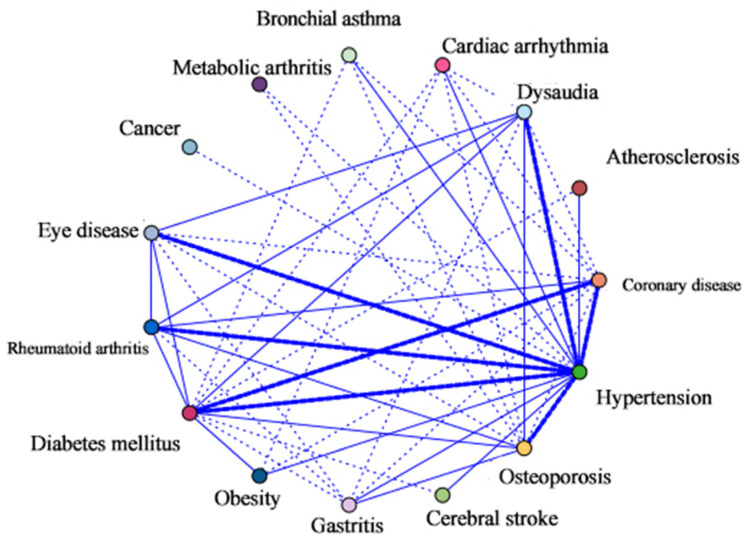
Web graph of association between chronic diseases.

The results of visualizing the influence of each node, representing a chronic disease, in the network are shown in Figure 2, with the inclusion criteria for chronic diseases at a prevalence of >1%. The network analysis demonstrated a strong link between hypertension and six kinds of chronic diseases: CAD, DM, a hearing impairment, eye disease, rheumatoid arthritis, and osteoporosis. In addition, there was also a strong link between DM and CAD. Moderate links included the combination of osteoporosis with rheumatoid arthritis, gastritis, DM, and a hearing impairment. Hypertension was liked with stroke, gastritis, obesity, bronchial asthma, arrhythmia, and atherosclerosis. In addition, diabetes with obesity, a hearing impairment, eye disease, and rheumatoid arthritis. Beyond that, the rest of the combinations were weak links. Therefore, the more prevalent an individual chronic disease, the more likely it contributes to multimorbidity combinations.

#### 3.2.2. Association Rules Mining Analysis

Given that network analysis only represents dyadic disease combinations, we further introduced ARM analysis to analyze the dyadic and triadic disease combinations. Under the conditions of min-support = 1.5%, min-confidence = 30%, the six association rules of multinomial chronic diseases and six of the ternary ones were selected. Table 2 shows that the probability for DM and HTN was 13.23%, CAD and DM was 6.13%, and osteoporosis and HTN was 4.48%. Among the respondents with two coexisting chronic diseases, HTN and DM were the most common multinomial combinations. In the triadic disease combination patterns, the probability for CAD-HTN-DM was 2.81%, rheumatoid arthritis-HTN-osteoporosis was 2.26%, osteoporosis-HTN-rheumatoid arthritis was 1.73%, osteoporosis-HTN-hearing impairment was 1.73%, and osteoporosis-HTN-DM was 1.73%. The multimorbidity patterns were led by hypertension and other chronic diseases, which was consistent with the findings of the network analysis. For example, ten multimorbidity patterns associated with hypertension were found in the older population.

### 3.3. Univariate Analysis of Factors Underlying Multimorbidity

The chi-square test showed statistically significant differences in the associated factors of multimorbidity in personal innate characteristics (age, family history, and BMI), behavioral characteristics (physical activity levels and medication adherence), family and social network (marital status and living arrangement), socioeconomic characteristics (education level), and three factors included in the macro-environmental characteristics (residence, basic endowment insurance, and basic medical insurance). However, no significant difference was found in the gender, smoking status, drinking status, and family structure of multimorbidity among older adults (Table 3).

### 3.4. Hierarchical Multiple Logistic Regression Results

A hierarchical multiple logistic regression analysis was performed to reveal the factors associated with suffering multimorbidity among the older adults. Multimorbidity was defined as a dependent variable in the logistic regression model.

In a multi-layered framework, Table 4 shows the results of hierarchical regression for multimorbidity. The personal innate characteristics variables were entered into Model 1. Although older adults aged 70 and above (70~ OR: 1.46, 95% CI: 1.22–1.74; 80~ OR: 1.56, 95% CI: 1.23–1.99) and those with a family history (OR: 2.22, 95% CI: 1.73–2.86) were found to be more likely to have multimorbidity and this model could significantly predict multimorbidity for older adults (*p* < 0.001), the explanatory power of 2.9% was not satisfactory. With the addition of variables for personal innate and behavioral characteristics, Model 2 shows that besides older age and the presence of a family history, low to moderate physical activity levels (low OR: 1.54, 95% CI: 1.23–1.92; moderate OR: 1.42, 95% CI: 1.16–1.73) and a low to moderate medication adherence (low OR: 1.42, 95% CI: 1.13–1.79; moderate OR: 0.56, 95% CI: 0.45–0.69) were significantly associated with suffering from multimorbidity. The explanatory power of Model 2 increased by 8.1% (*p* < 0.001) compared to Model 1. Based on Model 2, the variables for family and social network, including the marital status and living arrangements, were entered into Model 3. However, only widowhood was significantly related to multimorbidity (OR: 1.54, 95% CI: 1.23–1.93), while other factors were not significantly related to multimorbidity, and had an explanatory power of 8.8% (*p* < 0.001). In Model 4, older adults with elementary and below education were more likely to experience multimorbidity (OR: 1.42, 95% CI: 1.05–1.92), while other factors were insignificant. The explanatory power of this model increased to 10.1% (*p* < 0.001). In addition to the above dimensions with significant differences in Model 4, of the macro-environment variables, no variables were newly significant in Model 5. In contrast, the R^2^ value (10.8%) of Model 5 was the highest of the five models (*p* < 0.001). The Hosmer–Lemeshow test showed a good fit for the regression model (*p* = 0.448 > 0.05).

## 4. Discussion

The current study focused on the prevalence, patterns, and determinants of multimorbidity in older adults based on the health-ecological model. The results of the study show that the prevalence of multimorbidity among the older population in our study was 20.95%, higher than the rate reported in the United Kingdom (19.0%) [40] and Nepal (14.0%) [41], generally consistent with the prevalence reported in the Canada Community Health Survey (27.4%) [26], but lower than that of a study from Shandong, China (34.7%) [42]. These disparities can be explained by different sociodemographic structures and disease patterns [43]. In addition, the previous prevalence and patterns of multimorbidity may not be comparable due to differences in the sampling methods, sampling size, and number of chronic diseases enrolled. A systematic review that computed a pooled estimate of multimorbidity suggested that the reliability of the prevalence of multimorbidity studies may be decreased if less than 12 chronic diseases were included in the sample population [36]. Therefore, 25 chronic diseases were included in this study, resulting in more reliable results. To date, although many scholars have worked on the prevalence and associated factors of multimorbidity in different subgroups, many studies have been conducted in Western countries [16,40] and the health-ecological factors, such as the behavioral traits, lifestyle, and social context of Chinese populations are different compared to Western populations [44,45], so the etiological evidence from Western populations may not be applicable to Chinese populations [46].

The study also pointed out the patterns of multimorbidity in older adults. The results of the network analysis can show the possible association between chronic diseases. In this study, HTN was strongly linked with CAD, DM, a hearing impairment, eye disease, rheumatoid arthritis, and osteoporosis, while DM was strongly correlated with CAD. This is consistent with a systematic review of the disease patterns in the elderly in high-income countries, which noted that HTN, dyslipidemia, pain disorders, DM, and arthritis were very common in older adults [1]. To give more insight into the multimorbidity patterns, we also use ARM to represent the chronic diseases’ linkages. In the analysis of the ARM, the highest number of association rules related to the HTN was obtained, followed by DM. Similar trends were observed in the multi-countries research, revealing that the diseases most commonly coexisted with HTN and DM [10,47]. It is noticeable that the top two chronic diseases in the elderly were HTN and DM, which showed that these two chronic diseases not only have a high prevalence in the older population, but also have extensive associations between these two diseases and other diseases. Of the eight triadic disease combinations, five were associated with HTN or DM, and three showed a combination of HTN and two other diseases (e.g., osteoporosis-HTN-rheumatoid arthritis). These disease combinations could be named the cardio-metabolic pattern, cardio-degenerative pattern, and metabolic-degenerative pattern. It had been established in previous studies that the above multimorbidity pattern had similar risk factors and pathophysiology [48,49,50]. It could be hypothesized that if individuals are affected by HTN or DM, they are more likely to accumulate other metabolic or degenerative morbidities, such as high cholesterol, CAD, osteoporosis, etc. From a programmatic perspective, these findings highlight the urgent need for effective prevention and management strategies for individuals affected by multimorbidity [13,51]. Therefore, it is essential to pay more attention to the risk factors of the above-mentioned chronic diseases at the early stage of their development and strengthen the screening and diagnosis of multimorbidity for prevention.

The findings of the hierarchical multiple logistic regression analysis showed that the final model was interpreted more strongly than the original model, from Model 1 to 5, for multimorbidity. The view that multimorbidity is determined by multiple factors associated with personal traits and the macro environment [27] was verified once again.

At the personal innate layer, personal traits are the most direct contributors to multimorbidity. Many other factors, including lifestyle behavior, interpersonal networks, and macro-environment, ultimately influence the multimorbidity by acting on personal innate traits. It is well-established that the risk of developing chronic disease, especially multimorbidity, increases with age. Similar findings are also reported in the study by Hien et al. [52] that the prevalence of multimorbidity in older adults aged 60–69 years was 59.0%, and 71.8% among participants aged 70 years and above because older adults were exposed to risk factors for more extended periods as they age. A family history is considered to be the most significant risk factor for multimorbidity. Some studies have reported an association between family history and the risk of chronic disease [53,54]. In previous studies, obesity was a significant predictor of multimorbidity and increased the risk of developing specific multimorbidity clusters, such as cardio-metabolic patterns [55]. Interestingly, at the lifestyle behavior layer, our study found that a low medication adherence was a risk factor, while a moderate medication adherence was a protective factor for developing multimorbidity. The former was consistent with previous views [56], while the latter was probably due to our data being cross-sectional and could not determine causality between the variables, whereas older people with a high medication adherence tended to be those who were already suffering from disease or even multimorbidity [57], which is consistent with Domino’s study, which concluded that there was a lower non-adherence among patients with multimorbidity [58]. Older adults with lower levels of physical activity are more likely to suffer from multimorbidity. Christine et al. [59] showed that there was an inverse association between physical activity and multimorbidity among seniors aged ≥65 years, and this association was easier to be found in studies that include more than ten chronic diseases [60]. At the family and social network layer, widowhood was a risk factor for multimorbidity. It was consistent with previous studies [61], which indicates that support from a spouse is the most direct way for older adults to receive social support, while widowed older adults who suffer from psychological frustration and a smaller social network, are more likely to be multimorbidity. At the socioeconomic characteristic layer, having a primary education and below was considered to be risk factors for multimorbidity. The association between lower education and multimorbidity is unsurprising, given the well-established evidence that the knowledge and awareness of chronic disease prevention and the control of less educated people may be even lower, increasing the risk of developing multimorbidity [62]. At the macro-environmental characteristic layer, our study did not find an impact of the policy environment on older people with multimorbidity. However, the multimorbidity detection rate was higher among those without basic endowment insurance and medical insurance, suggesting that the policy environment may have an impact at the macro level. However, most policies are generally designed to assess the health of the population and cannot be defined at the individual level, so it may not be possible to use the macro environment to analyze their impact on individuals with multimorbidity accurately. However, this does not mean that macro-environmental factors are not necessary. On the contrary, many factors influence multimorbidity, and the results of this study reinforce that both proximal and distal factors should be considered.

### 4.1. Suggestions

The growing prevalence and varying patterns of multimorbidity demonstrate an urgency for the healthcare system to cope with the inescapable health threats and socioeconomic burdens. The implication reported in this study is helpful to guide future researches with the aim of promoting comprehensive multimorbidity prevention and control. As multimorbidity patients are exposed to more healthcare services and economic expenses, it is time for the healthcare system to shift from a single chronic disease model to a new financing and resource allocation model to manage multimorbidity more effectively [63]. Furthermore, clinical guidelines need to be developed based on the multimorbidity model, since figuring out the pattern in which diseases are associated with one another throughout individuals and knowing how frequently these diseases appear will bring about a better understanding of multimorbidity [24].

In 2009, China began to rebuild its primary healthcare system by strengthening the service capability of community health centers [2]. It is essential to provide preventive services to the whole population by utilizing health promotion and health education to guide residents to adopt healthy lifestyle behaviors and build a healthy and supportive environment, implementing the “Healthy China 2030” blueprint from all perspectives of the ecological model, and thus preventing the occurrence and development of multimorbidity.

### 4.2. Implications and Limitations

The results of the current study have important practical implications. One of the important research implications of this study may be that it is the first study to construct a hierarchical multiple logistic regression model to explore potential factors associated with multimorbidity, based on a health ecology framework. A multidimensional exploration of the influencing mechanisms of multimorbidity could be useful in revealing the link between the allocation of social resources and multimorbidity.

In spite of the valuable implication it offers, this study has some limitations. First, there were recall deviations and reporting deviations due to the cross-sectional design. Second, although there are 24 chronic diseases included in this study, only the elderly in Taiyuan, Shanxi Province, were surveyed. Second, the three Model 5 variables in the hierarchical logistic regression were not significantly associated. It is possible that more variables regarding social policies should be measured in further studies. Third, current survey did not collect information on the duration and severity of chronic diseases. In the future, further prospective cohort studies could be used to explore the factors influencing multimorbidity and their causal relationships, confirming and complementing the findings. Moreover, to explore the mediating and moderating relationships between the multidimensional factors and multimorbidity.

## 5. Conclusions

The main conclusion of this study are as follows: (1) multimorbidity is prevalent in older adults. The pattern of multimorbidity is dominated by dyadic and triadic, with a high co-occurrence of chronic diseases such as HTN, DM, rheumatoid arthritis, CAD, and hearing impairment. (2) Multimorbidity is prevalent and disproportionally distributed across varying personal innate-, behavioral-, family and social networks-, socioeconomic-, and macro-environmental characteristics among older adults. (3) Proximal and distal factors should be considered. A multi-level “individual-community-government” system management strategy should be established to manage the multimorbidity of older patients, older adults with multimorbidity, and specific disease association combinations which need to be given priority attention.

## Figures and Tables

**Table 1:** Prevalence of ten most prevalent chronic diseases and multimorbidity among older adults.

Chronic Disease	Number of Cases	Types of Chronic Diseases [N (%)]				Multimorbidity
	N (%)	1	2	3	≥4	N (%)
All participants	3637(100.0)	1742 (47.90)	522 (14.35)	167 (4.59)	73 (2.01)	762 (20.95)
HTN	1409 (38.7)	855 (60.7)	377 (26.8)	122 (8.7)	55 (3.9)	554 (39.3)
DM	481 (13.2)	213 (44.3)	165 (34.3)	70 (14.6)	33 (6.9)	268 (55.7)
Rheumatoid arthritis	226 (6.2)	99 (43.8)	65 (28.8)	37 (16.4)	25 (11.1)	127 (56.2)
CAD	223 (6.1)	87 (39.0)	72 (32.3)	42 (18.8)	22 (9.9)	136 (61.0)
Hearing impairment	213 (5.9)	86 (40.4)	59 (27.7)	44 (20.7)	24 (11.3)	127 (59.6)
Eye disease	199 (5.5)	72 (36.2)	67 (33.7)	36 (18.1)	24 (12.1)	127 (63.8)
Osteoporosis	163 (4.5)	57 (35.0)	43 (26.4)	34 (20.9)	29 (17.8)	106 (65.0)
Gastritis	114 (3.1)	51 (44.7)	27 (23.7)	22 (19.3)	14 (12.3)	63 (55.3)
Obesity	102 (2.8)	46 (45.1)	30 (29.4)	13 (12.7)	13 (12.7)	56 (54.9)
Bronchial asthma	77 (2.1)	33 (42.9)	22 (28.6)	10 (13.0)	12 (15.6)	44 (57.1)

HTN, Hypertension; DM, Diabetes mellitus; CAD, Coronary artery disease.

**Table 2:** Analysis results of multimorbidity dyads and triads by association rule.

Order	Consequent	Antecedent	Support (%)	Confidence (%)	Lift
1	HTN	DM	13.23	41.79	1.08
2	DM	CAD	6.13	45.71	1.18
3	DM	Eye disease	5.47	36.18	0.93
4	HTN	Osteoporosis	4.48	38.65	1.00
5	HTN	Digestive system disease	3.13	23.68	0.61
6	HTN	Obesity	2.81	31.38	0.81
7	DM	CAD, HTN	2.81	24.51	1.85
8	Osteoporosis	rheumatoid arthritis, HTN	2.26	21.95	4.90
9	rheumatoid arthritis	Osteoporosis, HTN	1.73	28.57	4.60
10	Hearing impairment	Osteoporosis, HTN	1.73	20.54	1.56
11	DM	Osteoporosis, HTN	1.73	20.54	1.56
12	HTN	Arrhythmia	1.60	34.48	0.89

HTN, Hypertension; DM, Diabetes mellitus; CAD, Coronary artery disease.

**Table 3:** Distribution of variables of older adults and group differences in multimorbidity and non-multimorbidity groups.

		Multimorbidity			
Characteristics	N (n = 3637)	Yes (n = 762)	No (n = 2875)	*χ* ^2^	*p* Value
**Personal innate characteristics:**					
**Gender**				3.680	0.055
Male	1864 (51.3)	367 (48.2)	1497 (52.1)		
Female	1773 (48.7)	395 (51.8)	1378 (47.9)		
**Age**				22.831	<0.001
60~	1816 (49.9)	322 (42.3)	1494 (52.0)		
70~	1315 (36.2)	315 (41.3)	1000 (34.8)		
80 and above	506 (13.9)	125 (16.4)	381 (13.3)		
**Family history**				40.472	<0.001
Yes	308 (8.5)	108 (14.2)	200 (7.0)		
No	3329 (91.5)	654 (85.8)	2675 (93.0)		
**BMI**				9.457	0.009
Lean (<18.6)	265 (7.3)	53 (7.0)	212 (7.4)		
Normal (18.5–23.9)	2060 (56.6)	398 (52.2)	1662 (57.8)		
Overweight (≥24)	1312 (36.1)	311 (40.8)	1001 (34.8)		
**Behavioral characteristics:**					
**Smoking status**				0.208	0.648
Yes	936 (25.7)	201 (26.4)	735 (25.6)		
No	2701 (74.3)	561 (73.6)	2140 (74.4)		
**Drinking status**				0.014	0.907
Yes	798 (21.9)	166 (21.8)	632 (22.0)		
No	2839 (78.1)	596 (78.2)	2243 (78.0)		
**Physical activity levels**				23.694	<0.001
Low	896 (24.6)	222 (29.1)	674 (23.4)		
Moderate	1505 (41.4)	335 (44.0)	1170 (40.7)		
High	1236 (34.0)	205 (26.9)	1031 (35.9)		
**Medication adherence**				120.971	<0.001
Low	1064 (29.3)	331 (43.4)	733 (25.5)		
Moderate	1932 (53.1)	277 (36.4)	1655 (57.6)		
High	641 (17.6)	154 (20.2)	487 (16.9)		
**Interpersonal network:**					
**Marital status**				22.831	<0.001
Married	2901(79.8)	566 (74.3)	2335 (81.2)		
Single	105(2.9)	20 (2.6)	85 (3.0)		
Divorced	42(1.2)	4 (0.5)	38 (1.3)		
Widowed	589(16.2)	172 (22.6)	417 (14.5)		
**Family structure**				1.871	0.171
Empty nest	1748 (48.0)	383 (50.3)	1365 (47.5)		
Non-empty nest	1889 (51.9)	379 (49.7)	1510 (52.5)		
**Living arrangement**				1.999	0.157
Living alone	657 (18.1)	151 (19.8)	506 (17.6)		
Living with others	2980 (81.9)	611 (80.2)	2369 (82.4)		
**Socioeconomic characteristics:**					
**Monthly income**				15.170	0.001
<1000 RMB	582 (16. 0)	154 (13.6)	434 (15.1)		
1000~3000 RMB	1666 (45.8)	529 (46.7)	1302 (45.3)		
>3000 RMB	1389 (38.1)	250 (32.8)	1139 (39.6)		
**Educational level**				41.573	<0.001
Elementary education and below	1376 (37.8)	365 (47.9)	1011 (35.2)		
Secondary education	1791 (49.3)	316 (41.5)	1475 (51.3)		
Higher education and above	470 (12.9)	81 (10.6)	389 (13.5)		
**Macro-environmental characteristics:**					
**Residence**				29.772	<0.001
Urban	2124(58.4)	379 (49.7)	1745 (60.7)		
Rural	1513(41.6)	383 (50.3)	1130 (39.3)		
**Basic endowment Insurance**				48.418	<0.001
Uninsured	1250 (34.4)	343 (45.0)	907 (31.5)		
Insured	2387 (65.6)	419 (55.0)	1968 (68.5)		
**Basic medical insurance**				4.433	0.035
Uninsured	365 (10.0)	92 (12.1)	273 (9.5)		
Insured	3272 (90.0)	670 (87.9)	2602 (90.5)		

**Table 4:** Hierarchical multiple logistics regression analysis of multi-layer factors related to multimorbidity.

Factors	Model 1		Model 2		Model 3		Model 4		Model 5	
	β	OR (95% CI)	β	OR (95% CI)	β	OR (95% CI)	β	OR (95% CI)	β	OR (95% CI)
**Personal innate characteristics:**										
**Age (Ref. 60–69)**										
70–79	0.38	1.46 (1.22–1.74) ***	0.37	1.45 (1.21–1.74) ***	0.30	1.35 (1.13–1.63) **	0.32	1.38 (1.15–1.66) **	0.33	1.39 (1.15–1.68) ***
80 and above	0.45	1.56 (1.23–1.99) ***	0.40	1.49 (1.17–1.91) **	0.27	1.32 (1.02–1.70) *	0.31	1.36 (1.05–1.76) *	0.34	1.41 (1.08–1.83) **
**Family history (Ref. No)**										
Yes	0.80	2.22 (1.73–2.86) ***	0.68	1.98 (1.53–2.56) ***	0.72	2.05 (1.58–2.66) **	0.74	2.09 (1.61–2.71) ***	0.75	2.11 (1.62–2.74) ***
**BMI (Ref. Normal)**										
Lean	0.02	1.02 (0.74–1.41)	−0.05	0.94 (0.67–1.31)	−0.08	0.91 (0.65–1.27)	−0.15	0.86 (0.62–1.21)	−0.20	0.82 (0.58–1.14)
Overweight	0.23	1.26 (1.06–1.50) **	0.23	1.26 (1.06–1.49) **	0.23	1.26 (1.05–1.50) **	0.22	1.24 (1.04–1.48) *	0.22	1.24 (1.04–1.48) *
**Behavioral characteristics:**										
**Physical activity levels (Ref. High)**										
Low			0.43	1.54 (1.23–1.92) ***	0.41	1.51 (1.21–1.89) ***	0.38	1.46 (1.16–1.83) **	0.34	1.41 (1.12–1.78) **
Moderate			0.34	1.42 (1.16–1.73) **	0.32	1.38 (1.12–1.68) **	0.32	1.38 (1.13–1.68) **	0.29	1.34 (1.09–1.64) **
**Medication adherence (Ref. High)**										
Low			0.35	1.42 (1.13–1.79) **	0.34	1.41 (1.12–1.78) **	0.29	1.34 (1.06–1.69) *	0.28	1.32 (1.05–1.68) *
Moderate			−0.58	0.56 (0.45–0.69) ***	−0.58	0.55 (0.44–0.69) ***	−0.62	0.54 (0.43–0.68) ***	−0.62	0.54 (0.43–0.68) ***
**Family and social network:**										
**Marital status (Ref. Married)**										
Unmarried					−0.09	0.91 (0.54–1.55)	−0.11	0.89 (0.53–1.54)	−0.09	0.91 (0.53–1.55)
Divorced					−0.87	0.42 (0.15–1.21)	−0.87	0.42 (0.15–1.21)	−0.84	0.43 (0.15–1.25)
Widowed					0.43	1.54 (1.23–1.93) ***	0.35	1.42 (1.12–1.78) **	0.35	1.42 (1.13–1.79) **
**Living arrangement (Ref. Living with others)**										
Living alone					−0.01	0.99 (0.79–1.24)	−0.05	0.95 (0.76–1.19)	−0.05	0.95 (0.76–1.19)
**Socioeconomic characteristics:**										
**Educational level (Ref. Higher education and above)**										
Elementary education and below							0.35	1.42 (1.05–1.92) *	0.39	1.48 (1.05–2.12) *
Secondary education							−0.05	0.95 (0.71–1.26)	−0.08	0.92 (0.69–1.23)
**Monthly income (Ref. >3000 RMB)**										
<1000 RMB							0.22	1.24 (0.95–1.62)	−0.01	0.99 (0.74–1.32)
1000–3000 RMB							0.19	1.22 (0.99–1.48)	0.13	1.14 (0.93–1.39)
**Macro-environmental characteristics:**										
**Residence (Ref. Urban)**										
Rural									0.08	1.08 (0.87–1.35)
**Basic endowment Insurance (Ref. Uninsured)**										
Insured									−0.27	0.76 (0.55–1.06)
**Basic medical insurance (Ref. Uninsured)**										
Insured									−0.12	0.89 (0.61–1.28)
**−2 Loglikelihood**	3657.751		3533.541		3514.315		3483.096		3464.779	
**χ^2^**	68.411		192.620		211.846		243.065		261.382	
**Sig**	0.000		0.000		0.000		0.000		0.000	
**Nagelkerke R^2^**	0.029		0.081		0.088		0.101		0.108	

* *p* < 0.05, ** *p* < 0.01, *** *p* < 0.001.

## Data Availability

Data are available, upon reasonable request, by emailing: cymtoemily@163.com.

## Supplementary Materials

The following supporting information can be downloaded at: https://www.mdpi.com/article/10.3390/ijerph192416756/s1.

## Abbreviations

BMI	Body mass index
HTN	Hypertension
DM	Diabetes mellitus
CAD	Coronary artery disease
