# Supplementary material for: Patterns and Determinants of Multimorbidity in Older Adults: Study in Health-Ecological Perspective
Source: Int J Environ Res Public Health. 2022 Dec 14;19(24):16756. doi: 10.3390/ijerph192416756 (PMC9779369; doi:10.3390/ijerph192416756)
Supplement: Supplementary file 1 [file ijerph-19-16756-s001.zip › ijerph-2085005-supplementary.pdf]

Survey of disease conditions (with diagnoses by a doctor)

The investigator is asked to rate the diagnosed chronic disease and fill in the severity of the disease (0, 1, 2, 3, 4) None = 0: There is no diagnosis of the disease or no such problem. Mild = 1: A diagnosis of the disease exists, but the disease does not affect the normal functioning of daily life and does not require special treatment. Moderate = 2: A condition that requires regular daily medication to control. Severe = 3: A condition that requires a combination of three or more medications or treatments other than first-line medication, combined with a condition that significantly limits the function of daily living. Very severe = 4: Severe limitation of daily functioning, chronic disease that is only partially controlled in terms of symptoms or severity despite maximum medication.

Any chronic disease	A=Yes B=No				
	None	Mild	Moderate	Severe	Very heavy
1. Coronary artery disease	0	1	2	3	4
2. Atherosclerosis	0	1	2	3	4
3. Arrhythmias	0	1	2	3	4
4. Hypertension	0	1	2	3	4
5. High cholesterol	0	1	2	3	4
6. Chronic bronchitis	0	1	2	3	4
7. Asthma	0	1	2	3	4
8. Cataracts	0	1	2	3	4
9. Glaucoma	0	1	2	3	4
10. Chronic gastritis	0	1	2	3	4
11. Chronic obstructive pulmonary disease	0	1	2	3	4
12. Chronic hepatitis	0	1	2	3	4
13. Chronic nephritis	0	1	2	3	4
14. Rheumatic arthritis	0	1	2	3	4
15. Gout	0	1	2	3	4
16. Osteoporosis	0	1	2	3	4
17. Sciatica	0	1	2	3	4
18. Stroke	0	1	2	3	4
19. Alzheimer's disease	0	1	2	3	4
20. Mental/emotional disorders	0	1	2	3	4
21. Diabetes mellitus	0	1	2	3	4
22. hyperthyroidism or hypothyroidism	0	1	2	3	4
23. Malignant tumors	0	1	2	3	4
24. Chronic disease anemia	0	1	2	3	4
